# Terror Management Theory and the COVID-19 Pandemic

**DOI:** 10.1177/0022167820959488

**Published:** 2021-03

**Authors:** Tom Pyszczynski, McKenzie Lockett, Jeff Greenberg, Sheldon Solomon

**Affiliations:** 1 University of Colorado at Colorado Springs, Colorado Springs, CO, USA; 2 University of Arizona, Tucson, AZ, USA; 3 Skidmore College, Saratoga Springs, NY, USA

**Keywords:** terror management theory, death anxiety, existential psychology, mental health, self-esteem, COVID-19, social unrest

## Abstract

Terror management theory is focused on the role that awareness of death plays in diverse aspects of life. Here, we discuss the theory’s implications for understanding the widely varying ways in which people have responded to the COVID-19 pandemic. We argue that regardless of whether one consciously believes that the virus is a major threat to life or only a minor inconvenience, fear of death plays an important role in driving one’s attitudes and behavior related to the virus. We focus on the terror management theory distinction between proximal defenses, which are activated when thoughts of death are in current focal attention and are logically related to the threat at hand, and distal defenses, which are activated when thoughts of death are on the fringes of one’s consciousness and entail the pursuit of meaning, personal value, and close relationships. We use this framework to discuss the many ways in which COVID-19 undermines psychological equanimity, the diverse ways people have responded to this threat, and the role of ineffective terror management in psychological distress and disorder that may emerge in response to the virus.

Although there are many disturbing aspects of the COVID-19 pandemic, from the perspective of terror management theory (TMT; Greenberg et al., 1986; Solomon et al., 2015), the enormous death toll and highly contagious nature of the virus play especially important roles in spawning the diverse forms of turmoil that have resulted from this crisis. We argue that the salience of death brought on by COVID-19 plays a central role in driving the attitudes and behavior of even those who believe that the dangers of the virus have been vastly exaggerated. TMT is focused on the pervasive role death awareness plays in human affairs. Much of what has been learned from the past 35 years of research applying TMT to diverse aspects of human behavior is directly relevant to understanding responses to the pandemic, especially the distinction between *proximal defenses*, which are directly focused on the problem of death, and *distal defenses*, which bear no logical relationship to death but enable people to construe themselves as valuable contributors to a meaningful, significant, and permanent universe (Pyszczynski et al., 1999). In this article we discuss how the salience of death inherent in COVID-19 influences diverse reactions to this pandemic.

## Terror Management Theory

TMT (Greenberg et al., 1986; Pyszczynski et al., 2015) posits that an inherent consequence of humankind’s sophisticated cognitive abilities is awareness of the inevitability of death. Awareness of death in an animal with an inherent proclivity for self-preservation gives rise to an ever-present potential for existential terror. This potential for terror is managed by an anxiety-buffering system consisting of *cultural worldviews, self-esteem*, and *close interpersonal relationships*. Cultural worldviews are shared beliefs about reality that provide answers to basic questions about life, standards for valued behavior, and the promise of *literal* or *symbolic immortality* to those who live up to these standards. Literal immortality beliefs provide hope that life will continue after physical death, as exemplified by afterlife concepts such as heaven, reincarnation, or joining with ancestral spirits. Symbolic immortality comes from contributing to something greater than oneself that will continue long after one has died, such as a family, nation, or the memories of others. Self-esteem is a sense of personal value that results from believing that one is living up to the standards of one’s cultural worldview. Close relationships provide consensual validation of one’s worldviews and self-esteem needed to maintain confidence in them, as well as providing security in their own right (Mikulincer et al., 2003). TMT posits that people manage the potential for anxiety inherent in awareness of the inevitability of death by maintaining faith in their cultural worldviews, self-esteem, and close relationships; these anxiety-buffering systems mitigate existential terror by imparting a sense that one is a person of value living in a meaningful world (for a more thorough presentation of these ideas, see Solomon et al., 2015).

Research has supported a network of converging hypotheses derived from TMT. This research shows that (1) reminders of death (*mortality salience*) increase commitment to one’s worldview, self-esteem, and relationships, and increase defense of these entities when threatened; (2) bolstering self-esteem, worldview, or relationships makes one less prone to anxiety and anxiety-related behavior in response to threats; (3) threats to worldview, self-esteem, and relationships increase the accessibility of death-related thoughts; and (4) self-esteem striving, cultural worldview defense, or affirming close relationships in response to mortality salience reduce death thought accessibility and the need for further terror management defenses; this suggests that the three components of the anxiety buffer are psychologically interchangeable (Hart et al., 2005). For a recent review of the TMT literature, see Pyszczynski et al. (2015). Meta-analyses have found strong evidence that reminders of death increase commitment to one’s worldview (Burke et al., 2010) and that threats to one’s worldview increase the accessibility of death-related thoughts (Steinman & Updegraff, 2015).

### Different Defenses for Conscious and Unconscious Death-Related Thoughts

TMT posits that people manage death anxiety with two distinct systems, referred to as *proximal and distal defenses* (Pyszczynski et al., 1999). When death-related thoughts are conscious (in current focal attention), proximal defenses are activated to suppress such thoughts or push death into the distant future by denying one’s vulnerability to things that could kill, or intending to engage in healthier behavior to ensure a longer life. However, when death-related thoughts are on the fringes of consciousness (no longer in focal attention but still highly accessible), people activate distal defenses focused on maintaining faith in their cultural worldview and enhancing self-esteem.

Conscious awareness of death requires defensive maneuvers that “make sense,” in that they imply that death is not a problem until many years in the distant future. But proximal defenses do little to quell anxiety stemming from the ultimate inevitability of death. These concerns are assuaged by distal defenses that are logically unrelated to death but imbue one’s life with meaning, value, and the promise of either literal or symbolic immortality. Research has shown that proximal defenses emerge shortly after reminders of death and that distal defenses emerge in response to death reminders only after a delay and distraction; however, distal defenses emerge immediately with no need for delay and distraction when death reminders are presented subliminally and thus bypass conscious attention. Research has also shown that distal defenses reduce the accessibility of death-related thoughts, which is presumably how they manage anxiety (see Arndt et al., 2002).

## Responses to the COVID-19 Pandemic as Terror Management Processes

The personal, social, economic, and political costs of the COVID-19 crisis are unprecedented. From the perspective of TMT, the root cause of all these problems is glaringly obvious—the risk of dying from the virus. Regardless of how contagious and lethal the virus ultimately turns out to be, or what one consciously thinks about it, the possibility of dying from it is highly salient and evident in ever-increasing death toll statistics, vivid images of overburdened hospitals and makeshift morgues, and the testimonials to victims of the virus, both famous and unknown. The deadly disease is spawned by an invisible pathogen that is conveyed by droplets expelled in the breath of its victims and thus might be lurking almost anywhere.

Since early March, media coverage of the pandemic in the United States and Europe has been virtually nonstop, interrupted only by coverage of a looming global economic collapse, lethal police violence against African Americans, and the protests and social upheaval in response to it. George Floyd’s death and related tragedies triggered a reinvigorated focus on social and economic injustice in American society, including peaceful protests and organized campaigns calling for police reform and greater support for the Black Lives Matter movement. However, media coverage has also highlighted the violence, vandalism, looting, and general disarray spawned by the protests and in response to them (Johnson, 2020; Kilgo, 2020). What has emerged from the co-occurrence of the pandemic and social upheaval is a constant barrage of threatening information. Moreover, it is impossible to visit a social media website without being inundated with new and often contradictory information on the virus. COVID-19 thus poses a ubiquitous dramatic reminder of vulnerability and death.

Because of its potential lethality, attempts to stem the tide of the virus or “flatten the curve” have led to closing many businesses and public venues and, consequently, led to loss of income and jobs, falling stock market values, general economic chaos, and social isolation, all of which seriously undermined major resources for managing the potential terror of death (FitzGerald et al., 2020). Public gatherings of all kinds were initially prohibited and then later strictly regulated, creating a void in personal contacts and near-total isolation for some (Banerjee & Rai, 2020). Information provided by governments, scientists, and the health care community has been confusing and sometimes contradictory, with partisan media outlets exacerbating the problem by providing narratives tailored to their constituents and critical of those with different ideological affiliations (Bermejo et al., 2020; Jurkowitz & Mitchell, 2020). These side effects of the pandemic seriously undermined all three components of the anxiety-buffering system that people use to maintain equanimity. The world has suddenly become an even more chaotic, confusing, and hostile place, in which death lurks around every corner, and people struggle to maintain meaning and self-esteem. People are living with the very real threat of death from the pandemic, combined with challenges to their worldviews, loss of jobs, impediments to career goals, and isolation from friends and family who normally validate one’s significance. From a TMT perspective, it is currently far more difficult for virtually all of us to manage the terror of death.

### Terror Management Health Model

People have responded to the pandemic in a wide variety of ways, some rational and some less so, some adaptive, and some destructive. The terror management health model (Goldenberg & Arndt, 2008) applies TMT and the distinction between proximal and distal defenses to health-related behavior. It suggests that thoughts of death can increase either motivation for healthy behavior or denial and avoidance when people are consciously focusing on them. However, when such thoughts are on the fringes of consciousness, they increase behavior oriented toward maintaining self-esteem and faith in one’s cultural worldview, which could either facilitate or undermine health. We now consider some of the ways in which people employ proximal and distal tactics to cope with COVID-19.

#### Proximal Defenses

TMT posits that when thoughts of death are in current focal attention, people attempt to remove them from their consciousness. This can entail simple suppression of such thoughts, denial of the threat, or engaging in behavior to reduce one’s vulnerability. Given the high level of media coverage, the changes in daily life that provide a constant reminder of the pandemic, and the extent to which virus-related concerns dominate conversations and media reporting, completely avoiding the issue is impossible. But there is evidence of increases in diversion-seeking behavior, such as alcohol consumption (Furnari, 2020), excessive eating (Ammar et al., 2020), and binge-watching television (Dixit et al., 2020).

Another form of proximal defense involves minimizing one’s perception of the threat. This has taken the form of arguing that the virus is not nearly as contagious or lethal as health experts claim it to be (Srikanth, 2020), or that it is only lethal for the elderly or those already at-risk of dying from other diseases (Fox et al., 2020). Others have trivialized the virus by comparing it to common illnesses such as the seasonal flu (Ritter, 2020), focusing on other common causes of death (McGinty, 2020), or viewing the publicity given the pandemic as originating in a politically motivated conspiracy (Romano, 2020). When sky-rocketing death statistics and vivid instances of contagion and mortality in the media make it hard to deny the problem outright, people sometimes claim that death rates are inflated to increase the funding hospitals receive (Nunez, 2020), or to bolster the aforementioned conspiracy to damage government leaders (Brown, 2020; Romano 2020).

Another, likely more adaptive, form of proximal defense against COVID-19 is to follow the prescriptions for avoiding infection provided by the medical community. This may be the most common proximal response to the pandemic; surveys suggest that 92% of people have followed guidelines for avoiding infection, to at least some extent (Altman, 2020). Most people have engaged in some form of social distancing, increased sanitation practices such as hand washing and cleaning surfaces, wore masks in public places, and done other things to stay healthy (Eanes, 2020). But the economic and social effects of these measures interfere with feelings of value and connection with the world, the core way in which we distally quell concerns about our mortality.

#### Distal Defenses

Despite its ubiquitous nature, thoughts of the virus are not *always* the focus of conscious thought. This would be too disturbing for most people to bear and could lead to the emergence or exacerbation of psychological disorders. In addition, the proximal defenses that people employ are likely to be at least somewhat effective in removing thoughts of the pandemic from our consciousness. The bulk of the TMT literature suggests that distal defenses focused on affirming one’s cultural worldview and maximizing self-esteem emerge when thoughts of death are highly accessible but not in focal attention; given the potential consequences of the virus and the enormous amount of attention the pandemic has attracted, this is likely to be the case for many people a great deal of the time.

Survey research provides clear evidence of a partisan divide in attitudes and behavior related to the virus. Liberals tend to view the virus as much more dangerous than conservatives, report considerably more personal distress about it, and have greater confidence in what scientists and medical professionals have to say about it (Funk et al., 2020; Ritter, 2020). Conservatives, on the other hand, view the virus as less dangerous and are more likely to assign blame to China and other foreigners and view the virus as part of a conspiracy to discredit Donald Trump (Romano, 2020). The rapid emergence of polarized liberal and conservative narratives about the virus illustrate the dynamic interplay between individual psychological forces and cultural worldviews that is central to the TMT analysis of the relationship between individual and cultural psychology.

This political divide is undoubtedly exacerbated by the accessibility of death-related cognition caused by the pandemic. Though surveys have documented this divide since before President Trump was elected, there is an even wider divergence regarding his overall handling of the pandemic (Bycoffe et al., 2020), attitudes toward those who have pushed back against some of his policies (Spangler, 2020), and Dr. Anthony Fauci, who was at one time the major voice for the administration but later voiced some disagreements (Brewster, 2020). A political divide is also evident in attitudes toward easing restrictions and reopening businesses and public places, with conservatives much more in favor of such policies than liberals. Whereas liberals tend to approve of societal restrictions to prevent the spread of the virus, conservatives tend to view them as unwarranted infringements on freedom and not worth the cost to the economy and individual incomes (Shepard, 2020). In many U.S. cities, protests against government restrictions were primarily attended by conservatives, some brandishing assault rifles and White nationalist symbols (Mauger, 2020; Perrett, 2020). Despite initial sentiment that “we’re all in this together,” the pandemic has become yet another domain for ideological division.

From a TMT perspective, reminders of death motivate people to affirm their worldviews, and political ideology is a central element of worldviews for many people. Though some studies show that mortality salience leads to a shift toward more conservative attitudes regardless of political orientation (Cohen et al., 2017; Landau et al., 2004), others show it leads to polarization, with conservatives endorsing more conservative attitudes and liberals endorsing more liberal ones (Kosloff et al., 2016). A meta-analytic review of this literature concluded that there is evidence for both tendencies, with the evidence being somewhat stronger for polarization (Burke et al., 2013). We are seeing this polarization playing out in both proximal and distal reactions to the threat of the virus. One current example of intensified reactions may be the powerful and sometimes violent protests in response to the killing of George Floyd. This was far from the first unjust killing of a Black person by the police, but it has clearly led to the most intense and widespread outrage and protests of any of them. Perhaps it is the final tragic straw that broke the proverbial camel’s back that occurred at a time when people had more time to engage due to shutdowns in response to the virus. But we argue that the background of death thought accessibility due to the pandemic probably intensified these reactions. Fueled by a greater need for terror management, many people jumped fervently onto this cause as a way to feel that they are doing something of value in their lives, when in reality their ability to feel that way has been so hampered by loss of jobs and income, social isolation, and difficulties in making sense of the tragedy and divisiveness that has emerged in the wake of the virus.

Meaning and significance derived from participating in these mass social protests may ironically *increase* death salience as protestors gather in large groups that exponentially increase their chance of exposure to the virus. Protests are also threatening, in that—though most have been peaceful—there is a lurking potential for violence with police and counter-protesters. Many people have suffered serious, life-altering injuries by “nonlethal weapons” used by police officers to control protests and riots; for example, some individuals have been permanently blinded after being shot with rubber bullets (Bauerlein & Calvert, 2020; Sheikh & Montgomery, 2020). Interestingly, the individuals who are risking their lives to protest racial inequality tend to hold the same political views as those who believe that social distancing should be practiced (Diamond, 2020; Nguyen, 2020). Thus, these individuals are in a precarious position, where bolstering one’s cultural worldview by protesting racial inequality involves directly, consciously putting oneself in danger of violence and disease. TMT suggests that affirming one’s worldview—along with one’s self-esteem and close relationships—maintains psychological well-being by buffering death-related thoughts by providing outlets for symbolic immortality. The current crisis raises the intriguing question of what happens when affirming one’s worldview involves putting oneself directly in harm’s way and, consequently, never fully removing the salience of death from one’s consciousness.

### Overwhelmed Anxiety Buffers and Psychological Disorder

Consistent with the theoretical writings of Becker (1973), Lifton (1979), and Yalom (1980), TMT suggests that when people are not effectively managing their existential terror by building a meaningful and purposeful life, death anxiety and maladaptive ways of dealing with that anxiety are the common result. Indeed, it has been argued that both death anxiety and ineffective or disrupted anxiety-buffer functioning are transdiagnostic vulnerability factors for psychological disorder (Iverach et al., 2014; Yetzer & Pyszczynski, 2018). If fear of death does indeed motivate the pursuit of meaning in life, self-esteem, and close relationships, then problems in managing death concerns exacerbated by the pandemic would leave people overwhelmed with anxiety and therefore more vulnerable to psychological disorder. Experimental research has shown that reminders of mortality exacerbate phobias, obsessive-compulsive behaviors, depressive affect, and anxiety (e.g., Finch et al., 2016; Menzies & Dar-Nimrod, 2017; Mikulincer et al., 2020; Strachan et al., 2007). This may help explain why a recent review found that the pandemic is associated with increased reports of anxiety, depression, and stress (Torales et al., 2020; Wang et al., 2020).

The COVID-19 pandemic might cause psychological distress in two ways that correspond with proximal and distal defenses against death-related thought. First, the pandemic has directly increased death anxiety by raising awareness of personal vulnerability; recent research shows that the pandemic has increased anxiety and fear regarding one’s physical well-being (Jungmann & Witthöft, 2020). In addition, maladaptive proximal defenses may entail harmful practices aimed at avoiding the virus; some people have gargled bleach and cleaning supplies to reduce their chances of catching the virus (Gharpure et al., 2020). People are also employing a variety of unhealthy distractions to shift their focus away from the threat of the virus, including opiate use (https://www.ama-assn.org/system/files/2020-06/issue-brief-increases-in-opioid-related-overdose.pdf) and gambling (https://www.marketwatch.com/story/online-poker-betting-hits-a-record-high-during-the-pandemic-2020-05-29).

The pandemic has also undermined distal defenses by hampering or eliminating the anxiety-buffering outlets that people typically rely on to believe that they are valuable contributors to a meaningful world. When people lose their jobs and cannot pursue their financial, educational, and career goals, they are losing important sources of self-esteem. Social relationships, which play such a major role in managing death fears, have also been hampered by the lockdown and social distancing measures. Single people looking for a potential life partner have largely had to put this pursuit on hold. COVID-19-related stress resulting from all of these aspects of the pandemic is associated with lower levels of meaning in life and life satisfaction (Trzebiński et al., 2020). Inadequate distal defenses are likely to affect the need for proximal defenses and vice versa. Increased death awareness associated with the threat of COVID-19 is difficult to successfully manage because COVID-19 has undermined access to many aspects of people’s anxiety buffers; compromised anxiety buffers leave people vulnerable to experiencing higher levels of death anxiety than usual.

How might one manage these overwhelmed death-related defenses? Understanding the existential threats associated with the pandemic and reflecting on the proximal and distal defenses one uses to cope with them may help people develop new coping skills in these unprecedented times. For individuals experiencing high levels of death and health anxiety, managing one’s engagement with virus-related information may help reduce explicit death anxiety. A recent study found that social media exposure during the pandemic is associated with poorer mental health as it likely contributes to the persistent salience of the virus and its mortality threat (Gao et al., 2020). Engaging in and acknowledging the efficacy of best practices for avoiding infection is another potentially useful strategy for reducing anxiety. Feelings of meaninglessness resulting from the loss of social relationships and self-esteem sources may be addressed by finding new sources of meaning, significance, and interpersonal connection; preferably ones that don’t increase the threat of contracting or spreading the virus. Reported increases in home-based hobbies such as baking bread or exercise have become popular as ways to derive a sense of meaning and value during the pandemic (VanDerWerff, 2020). Social events that have been cancelled have sometimes been redesigned as COVID-friendly occasions—for example, the increasing popularity of drive-in theaters; online education; outdoor activities, such as hiking, where one can socialize while maintaining distance; and virtual get-togethers for parties, weddings, and funerals. “The New Normal” has quickly become a cliché in light of the pandemic, yet it succinctly describes people’s adaptations for both avoiding the threat of death from the virus while still being able to pursue goals that give life meaning, value, and connection with others. Acknowledging one’s emotional distress in response to the pandemic may encourage more creative and constructive ways of coping with it.

## Conclusion: The Push and Pull Between Proximal and Distal Defenses

The tension between measures to keep us safe from this oft-deadly virus and the desire to reopen the economy and resume “normal” life can be viewed as a battle between proximal and distal defenses against death. Proximally, we want to forestall death and feel safe from it in the short term. Distally, we want to maintain the view that life is meaningful and that we are valuable contributors to that meaningful life. The fundamental dilemma is that measures that keep us safe in the moment often interfere with our ability to find meaning and significance in our lives. Both are important psychological concerns, and finding the right compromise to sufficiently meet both needs is the great challenge every culture is facing. One tragic example of this is that to keep hospitals and nursing homes safe, loved ones are often not allowed to be with their sicker or dying friends and family members. The result is people facing their own and their loved ones’ imminent death without the support systems that provide them with their deepest psychological security. Perhaps understanding these issues from the perspective of TMT can help societies determine the best versions of the many compromises with which they contend as they move forward with life in the face of the COVID-19 pandemic.
